# Early pancreatic volume reduction on CT predicts relapse in patients with type 1 autoimmune pancreatitis treated with steroids

**DOI:** 10.1186/s13023-016-0487-y

**Published:** 2016-07-28

**Authors:** Yoshinori Ohno, Teru Kumagi, Tomoyuki Yokota, Nobuaki Azemoto, Yoshinori Tanaka, Kazuhiro Tange, Nobu Inada, Hideki Miyata, Yoshiki Imamura, Mitsuhito Koizumi, Taira Kuroda, Yoichi Hiasa, on behalf of the EPOCH Study Group

**Affiliations:** 1 Gastroenterology and Metabology, Ehime University Graduate School of Medicine, Shitsukawa, Toon, Ehime 791-0295 Japan; 2 Center for Liver-Biliary-Pancreatic Diseases, Matsuyama Red Cross Hospital, Matsuyama, 790-8524 Ehime Japan; 3 Gastroenterology, Ehime Prefectural Central Hospital, Matsuyama, 790-0024 Ehime Japan; 4 Gastroenterology, Matsuyama Municipal Hospital, Matsuyama, 790-0067 Ehime Japan; 5 Internal Medicine, Saiseikai Imabari Hospital, Imabari, 799-1502 Ehime Japan; 6 Internal Medicine, Saiseikai Matsuyama Hospital, Matsuyama, 791-8026 Ehime Japan

**Keywords:** Autoimmune pancreatitis, Relapse factor, Pancreatic volume, Steroid therapy, CT

## Abstract

**Background:**

Type 1 autoimmune pancreatitis (AIP) is clinically characterized by a response to steroid therapy. Despite having a favorable prognosis, AIP has a high relapse rate and factors predicting relapse in AIP patients treated with steroids have not yet been established.

**Methods:**

A retrospective chart review was conducted of 32 newly diagnosed type 1 AIP patients who had undergone enhanced computed tomography (CT) pre- and post-steroid therapy.

**Results:**

Ten patients experienced relapse. Pancreatic volume was reduced significantly in all patients (pre-treatment volume, 88.5 ± 32.9 cm^3^ vs. post-treatment volume, 45.4 ± 21.1 cm^3^; *P* < 0.001), although the pre-treatment pancreatic volume did not differ between the relapse and non-relapse groups (92.6 ± 10.5 cm^3^ vs. 86.6 ± 7.1 cm^3^, *P* = 0.401). However, the post-treatment pancreatic volume was significantly greater in the relapse group than that in the non-relapse group (56.9 ± 6.3 cm^3^ vs. 40.2 ± 4.2 cm^3^, *P* = 0.008). Similarly, the percent reduction in pancreatic volume was significantly smaller in the relapse group than that in the non-relapse group (36.6 ± 4.7 % vs. 52.1 ± 3.2 %, *P* = 0.002). Multivariate analysis identified post-treatment pancreatic volume (HR, 1.04, 95 % CI: 1.01–1.08, *P* = 0.010) and percent reduction in pancreatic volume (HR, 0.87, 95 % CI: 0.79–0.94, *P* < 0.001) as predictive factors for relapse of type 1 AIP. A post-treatment pancreatic volume of 50 cm^3^ < (*P* = 0.009) and a percent reduction in the pancreatic volume of <35 % (*P* = 0.004) had a significantly high relapse rate. These data suggest that early pancreatic volume changes after steroid therapy may be a useful prognostic value, because type 1 AIP patients with a high post-treatment pancreatic volume or low pancreatic volume reduction showed significant relapse.

**Conclusions:**

Early pancreatic volume reduction on CT after steroid therapy indicates the therapeutic effects of steroids, and a low decrease in the pancreatic volume may be associated with a limited response that predicts future relapse in patients with type 1 AIP. Reduction of steroids in these cases must be observed carefully with consideration of immunomodulator use.

## Background

Type 1 autoimmune pancreatitis (AIP) is associated with the enlargement of the pancreatic parenchyma, abundant lymphoplasmacytic infiltration and fibrosis, and frequent elevations in the serum immunoglobulin (Ig)-G4 levels. Although the precise pathogenesis of AIP has not yet been determined, AIP is clinically characterized by a response to steroid therapy, and it has a favorable prognosis [1–6]. The remission rate of steroid-treated AIP is 98 %, which is significantly higher than 74 % of patients without steroid treatment [7]. However, many patients will experience disease relapse in type 1 AIP, and the relapse rate for this type is 15–64 %, according to various studies [7–10]. For most patients in previous reports, relapses occurred after steroid discontinuation [7, 11]. Kamisawa et al. reported that continued maintenance treatment with low-dose prednisolone for 6 months to 3 years is also recommended to prevent relapse in type 1 AIP [7]. Patients who resumed steroid treatment continued to respond favorably with a high remission rate. In addition, some patients with relapse were treated with an immunomodulator [9, 11]. Although there is general agreement that long-term steroid therapy is the ideal initial treatment for preventing disease relapse, the incidence of steroid-related side effects is a major concern. If predictive factors of relapse or non-relapse in patients with type 1 AIP exist, some patients may not require long-term steroid maintenance therapy, and the incidence of treatment-related side effects may decrease. However, factors that may predict relapse have not yet been established. In this context, the Ehime Pancreato-Cholangiology (EPOCH) Study Group conducted a retrospective study to identify the predictive factors of relapse in patients with type 1 AIP by focusing on the volume changes in the pancreas.

## Methods

### Patients

This study included 41 consecutive cases of type 1 AIP according to the clinical diagnostic criteria proposed by the Japan Pancreas Society [12] or the International Consensus of Diagnostic Criteria [13] at six gastroenterology clinics in Ehime (EPOCH Study Group), Japan, from January 2006 to March 2015. Patients who were on steroid therapy before the final diagnosis (*N* = 2), who were not given steroid therapy (*N* = 3), and who did not undergo post-treatment computed tomography (CT) after steroid therapy (*N* = 4) were excluded. Finally, this study included 32 newly diagnosed type 1 AIP patients who underwent enhanced CT pre- and post-treatment.

In 23 patients (71.9 %), an accompanying pancreatic malignancy was pathologically excluded by an endoscopic ultrasonography-guided fine needle aspiration biopsy (*N* = 22) or by surgical resection (*N* = 1). The median length of follow-up was 36 months (range, 3–107 months). Pre- and post-treatment data collection included demographics (i.e., age and sex), complications (e.g., jaundice and diabetes), markers associated with AIP (i.e., IgG and IgG4), extra-pancreatic lesions detected on CT, and extension of biliary stricture determined by magnetic resonance cholangiopancreatography or endoscopic retrograde cholangiopancreatography (i.e., intrapancreatic, extrapancreatic, and intrahepatic).

#### CT interval and methods of CT scan measurements

We measured the volumes of the pancreas as shown on enhanced CT obtained before and within 6 months (median, 1.6 months) after steroid therapy. Early-phase contrast-enhanced CT was used to measure the pancreatic volume, and the slice thickness was as follows: 5 mm (*N* = 29), 7 mm (*N* = 2), and 10 mm (*N* = 1). The Synapse® Imaging System (Fuji Film) was used to measure the pancreatic volume, which was calculated by summing the manually contoured area of the pancreatic outline of each CT slice. The splenic volume was measured by using the same methods as those for pancreatic volumetry.

#### Steroid therapy and disease relapse

The initial daily oral prednisolone dose was determined by body weight (0.6 mg/kg per day), and it was 30 mg/day in 27 patients (84 %). This dosage was administered for about 2 weeks and then was tapered gradually until a daily dose of 5–10 mg was reached, as per each physician’s decision. Maintenance steroid therapy was given to all patients, and steroid therapy was discontinued in 4 patients (range, 9–12 months). The median length of steroid therapy was 34 months. The relapsed group was defined as follows: reappearance of the symptoms with reappearance of pancreatic and/or extra-pancreatic abnormalities on imaging studies regardless of the serum IgG or IgG4 levels [7].

#### Statistical analysis

The data were analyzed using JMP 9.0 (SAS Institute). Differences and correlations in the pancreatic volume before and after steroid therapy were compared using the Wilcoxon signed-rank test. Receiver operating characteristic (ROC) curves were generated, and the cutoff was determined as the point in the ROC curve that maximized the value of sensitivity plus specificity. Cox proportional hazards regression was used to perform univariate and multivariate analyses of the predictors of disease relapse. Relapse-free survival rates were calculated using the Kaplan-Meier method. P-values <0.05 were considered significant.

## Results

### Clinical profiles

As shown in Table 1, a total of 32 newly diagnosed type 1 AIP patients were enrolled. Twenty-six patients (81.3 %) were male, and the median age was 63 years (range, 32–89 years). Patients presented with obstructive jaundice (*N* = 13), abdominal pain or discomfort (*N* = 13), worsening of diabetes (*N* = 2), and abnormal abdominal imaging (*N* = 4). Twenty-three patients (71.9 %) had diffuse pancreatic swelling and 13 (40.6 %) had jaundice. Patients had the following complications: bile duct stricture (*N* = 26, 81.3 %), all were intrapancreatic but one was intrahepatic; retroperitoneal fibrosis (*N* = 3, 9.4 %); and sialadenitis (*N* = 2, 6.3 %). Twenty patients (62.5 %) had diabetes: 12 patients preceded the diagnosis of AIP, and 8 patients were diagnosed simultaneously with the diagnosis of AIP. Relapse was observed in 10 patients (31.3 %, relapsed group), with a median duration of 5.0 months (range, 2.1–47.2 months). The reasons for relapse were as follows: symptoms and laboratory data suggestive of bacterial cholangitis with an intrapancreatic bile duct stricture (*N* = 6), acute pancreatitis (*N* = 3), and swelling of the pancreas and the emergence of retroperitoneal fibrosis (*N* = 1) (Table 1). The relapse rate in patients on maintenance therapy was significantly lower than that in those who discontinued maintenance therapy (23 % [7/30 patients] vs. 75 % [3/4 patients]; *P* = 0.033). Surgeries were performed in 2 patients during the observation period. Pancreatic cancer was suspected in one; however, this was not confirmed, and the other patient had intractable bacterial cholangitis. Finally, all patients survived, except one who died due to a cerebrovascular disease.

**Table 1:** Clinical findings with type 1 autoimmune pancreatitis

	Type 1 AIP (*N* = 32)
Age (years old)	63 (32–89)
Gender (male/female)	26 / 6
Imaging of pancreatic parenchyma	
Diffuse swelling	23 (71.9 %)
Elevated serum IgG4 level (≧135 mg/dl)	25 (78.1 %)
Obstructive Jaundice	13 (40.6 %)
Bile duct stricture	
Intrapancreatic	25 (78.1 %)
Extrapancreatic	0
Intrahepatic	1 (3.1 %)
Retroperitoneal fibrosis	3 (9.4 %)
Sialadenitis	2 (6.3 %)
Diabetes	20 (62.5 %)
Relapse of after steroid therapy	10 (31.2 %)

### Pancreatic volume change and a comparison of the clinical profiles of the relapse and non-relapse groups after steroid therapy

**Fig. 1:**
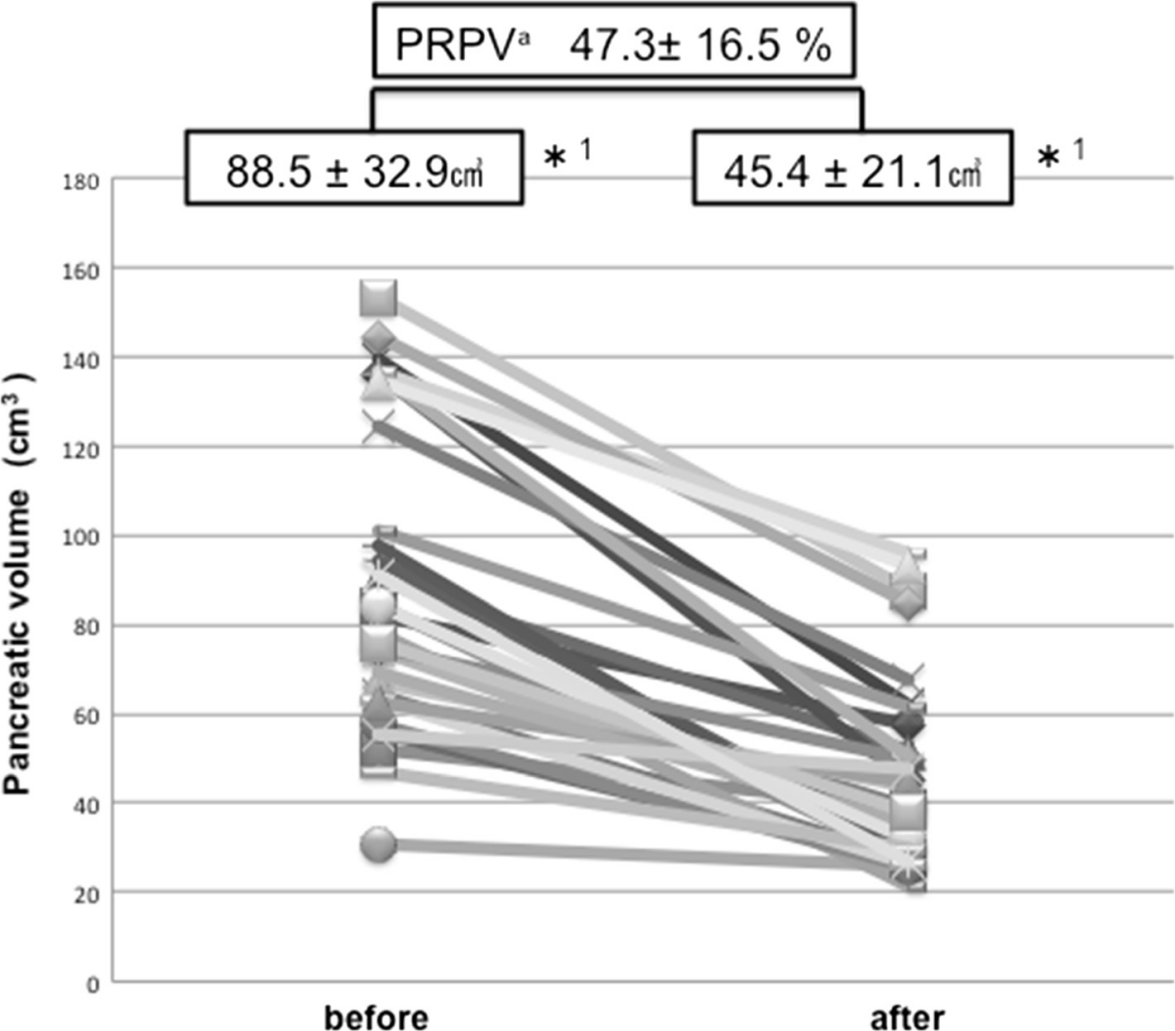
Comparison of the pancreatic volume change after steroid therapy. *^1^ < 0.001 by Wilcoxon signed-rank test. ^a^Percent Reduction in the Pancreatic Volume = 100 % - (pancreatic volume after steroid therapy/pancreatic volume before steroid therapy) × 100 %

**Fig. 2:**
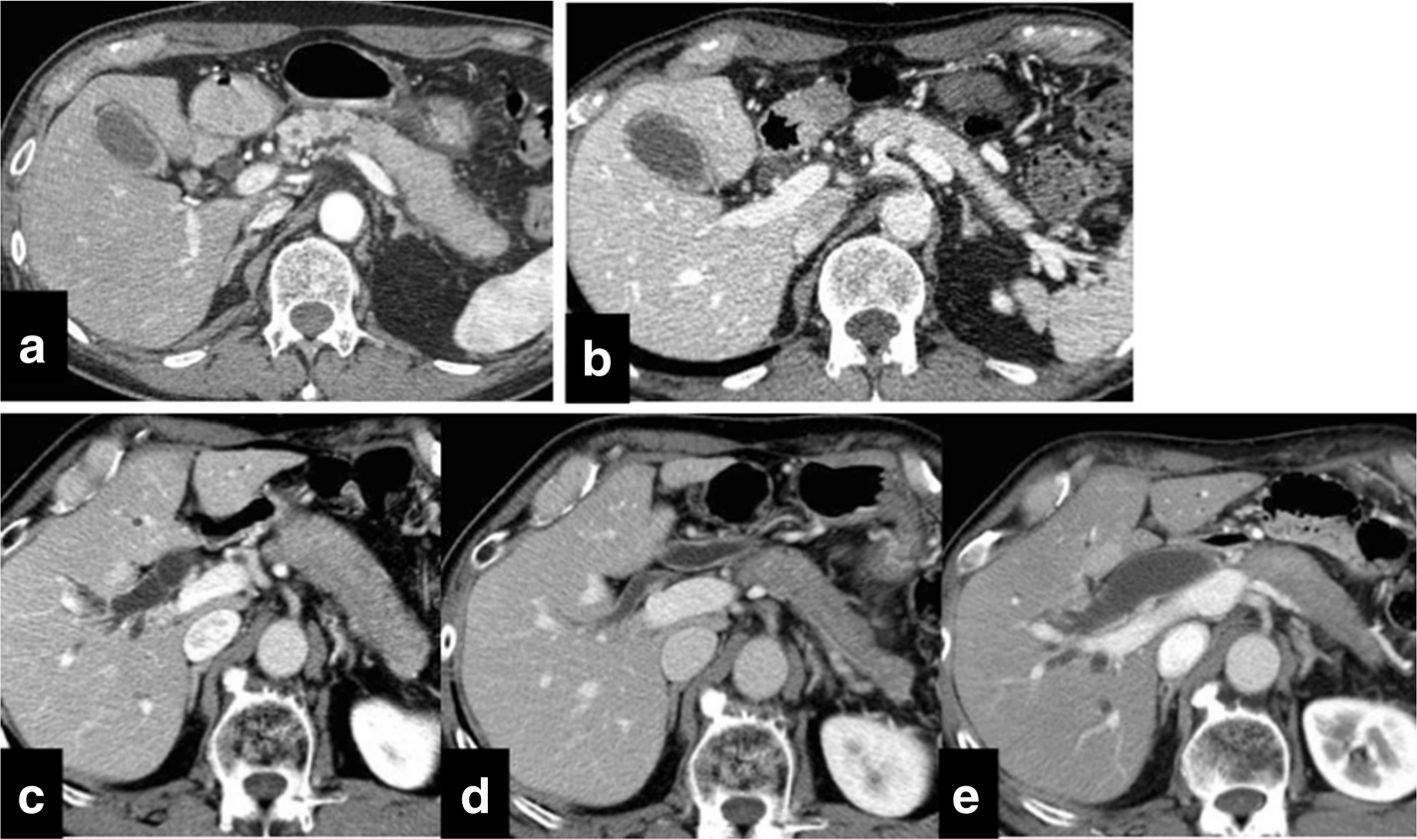
Abdominal computed tomography showing the pancreas in patients with autoimmune pancreatitis. The post-treatment pancreatic volume was 30.3 cm^3^, and the percent reduction in the pancreatic volume was 68.2 % in a patient who showed a favorable response without relapse before steroid therapy (**a**) and 2 weeks after starting steroid therapy (**b**). The pancreatic volume was not reduced in a patient who showed an unfavorable response with relapse. The post-treatment pancreatic volume was 51.6 cm^3^, and the percent reduction in the pancreatic volume was 42.3 % before steroid therapy (**c**) and after 2 months starting steroid therapy (**d**); at 47 months during relapse, dilatation of the biliary tree (**e**) was observed

**Fig. 3:**
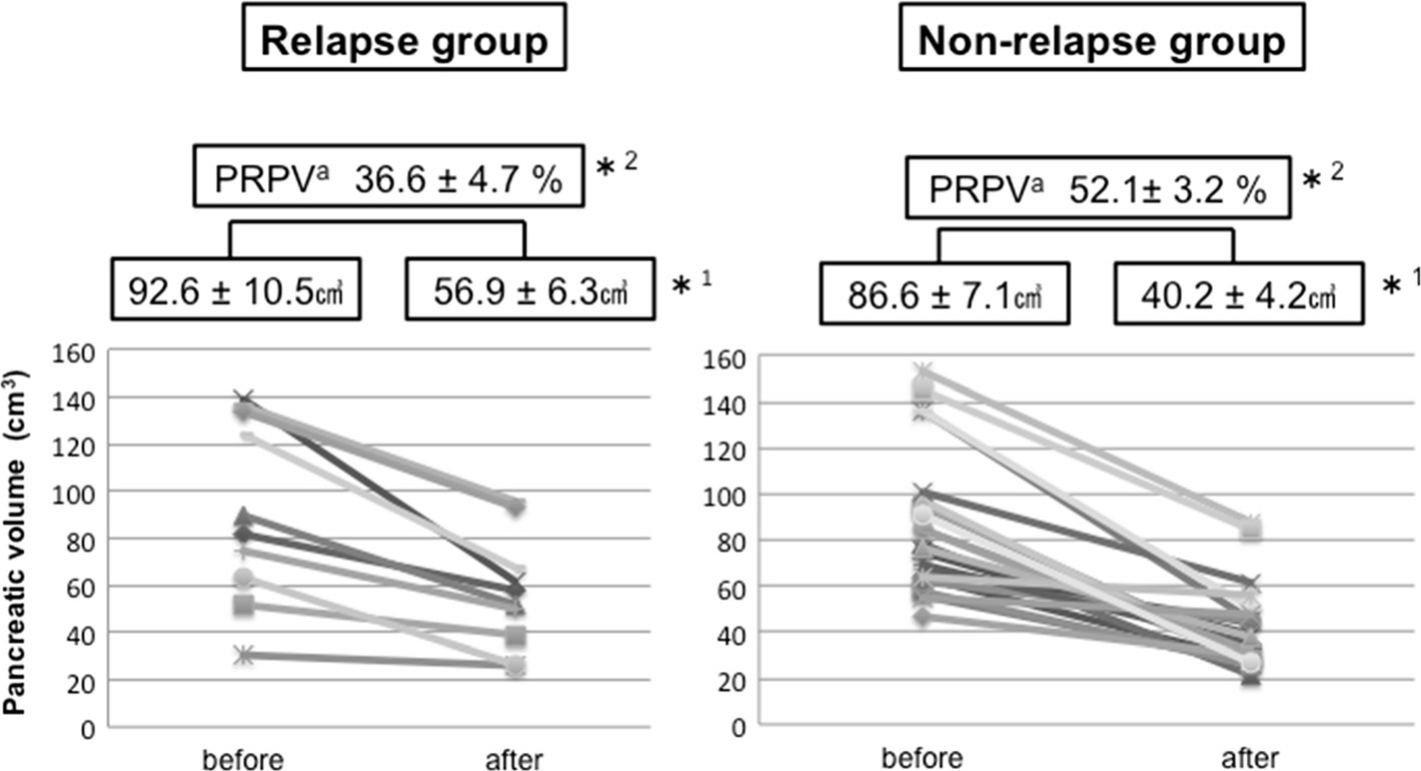
Comparison of pancreatic volume change in patients with or without relapse. *^1^ < 0.008, *^2^ < 0.002 by Cox proportional hazards regression. ^a^Percent Reduction in the Pancreatic Volume = 100 % - (pancreatic volume after steroid therapy/pancreatic volume before steroid therapy) × 100 %

In all patients, the pancreatic volume on CT was reduced significantly by steroid therapy (pre-treatment volume, 88.5 ± 32.9 cm^3^ vs. post-treatment volume, 45.4 ± 21.1 cm^3^; *P* < 0.001), with an average reduction of 47.3 ± 16.5 % (Fig. 1). When the relapse and non-relapse groups were compared, the pancreatic volume did not differ between the two pre-treatment groups (92.6 ± 10.5 cm^3^ vs. 86.6 ± 7.1 cm^3^, *P* = 0.401). However, the post-treatment pancreatic volume in the relapse group was significantly higher than that in the non-relapse group (56.9 ± 6.3 cm^3^ vs. 40.2 ± 4.2 cm^3^; *P* = 0.008). Similarly, the percent reduction in the pancreatic volume was significantly smaller in the relapse group than in the non-relapse group (36.6 ± 4.7 % vs. 52.1 ± 3.2 %, *P* = 0.002). Abdominal CT showed favorable and unfavorable responses to steroid therapy in patients with AIP (Fig. 2). The volume of the spleen on CT was also measured, and it was slightly reduced by steroid therapy (pre-treatment splenic volume, 134.7 ± 58.7 cm^3^ vs. post-treatment splenic volume, 116.1 ± 51.4 cm^3^, *P* < 0.001). However, when the relapse and non-relapse groups were compared, the volume of the spleen did not differ between the two pre-treatment groups. In univariate analysis, variables other than the post-treatment pancreatic volume and percent reduction in the pancreatic volume failed to show an association with relapse (Table 2, Fig. 3). Multivariate analysis used two models and included the post-treatment pancreatic volume (only in Model 1), percent reduction in the pancreatic volume (only in Model 2), diffuse pancreatic swelling, bile duct stricture, and serum IgG4. Finally, the post-treatment pancreatic volume (HR = 1.04, 95 % CI: 1.01–1.08, *P* = 0.010) and percent reduction in the pancreatic volume (HR = 0.87, 95 % CI: 0.79–0.94, *P* < 0.001) were identified as a predictive factor for relapse in AIP (Table 3). However, diffuse pancreatic swelling, bile duct stricture, and serum IgG4 were not identified as predictive factors.

**Table 2:** Comparison of the clinicopathologic characteristics in patients

Parameter	Relapse		
	(+) (*N* = 10)	(−) (*N* = 22)	*P* value
Age (years old)	61.6 ± 3.6	60.5 ± 2.4	0.799
Gender (male)	8	18	0.732
Imaging of pancreatic parenchyma (Diffuse)	8	15	0.897
IgG (mg/dl)	2020 ± 298	1877 ± 205	0.398
IgG4 (mg/dl)	525 ± 124	368 ± 84	0.483
Obstructive jaundice	4	9	0.560
Bile duct stricture^a^	8	17	0.795
Extra-pancreatic lesions^b^	2	4	0.988
Diabetes	5	15	0.268
Pre-treatment pancreatic volume (cm^3^)	92.6 ± 10.5	86.6 ± 7.1	0.401
Post-treatment pancreatic volume (cm^3^)	56.9 ± 6.3	40.2 ± 4.2	0.008*
Percent reduction of pancreatic volume (%)	36.6 ± 4.7	52.1 ± 3.2	0.002*
Pre-treatment splenic volume (cm^3^)	133.9 ± 18.9	135.0 ± 12.7	0.959
Post-treatment splenic volume (cm^3^)	116.5 ± 16.5	116.0 ± 11.1	0.836
Percent reduction of splenic volume (%)	13.5 ± 6.3	12.3 ± 4.2	0.616

^a^ Intrapancreatic bile duct stricture, except extrapancreatic and intrahepatic bile duct stricture

^b^ Intrahepatic bile duct stricture, retroperitoneal fibrosis, and sialadenitis

* < 0.05 by Cox proportional hazards regression analysis

**Table 3:** Results of multivariate analysis for disease relapse in patients with AIP

	HR	95 % CI	*P* value
Model 1			
Post-treatment pancreatic volume	1.04	1.01–1.08	0.010
Model 2			
Percent reduction in the pancreatic volume	0.87	0.79–0.94	<0.001

Data are based on Cox proportional hazards regression

Model 1: post-treatment pancreatic volume, diffuse pancreatic swelling, bile duct stricture (intrapancreatic bile duct stricture, except extrapancreatic and intrahepatic bile duct stricture), and serum IgG4

Model 2: percent reduction in the pancreatic volume, diffuse pancreatic swelling, bile duct stricture (intrapancreatic bile duct stricture, except extrapancreatic and intrahepatic bile duct stricture), and serum IgG4

### Predictive factors of relapse

**Fig. 4:**
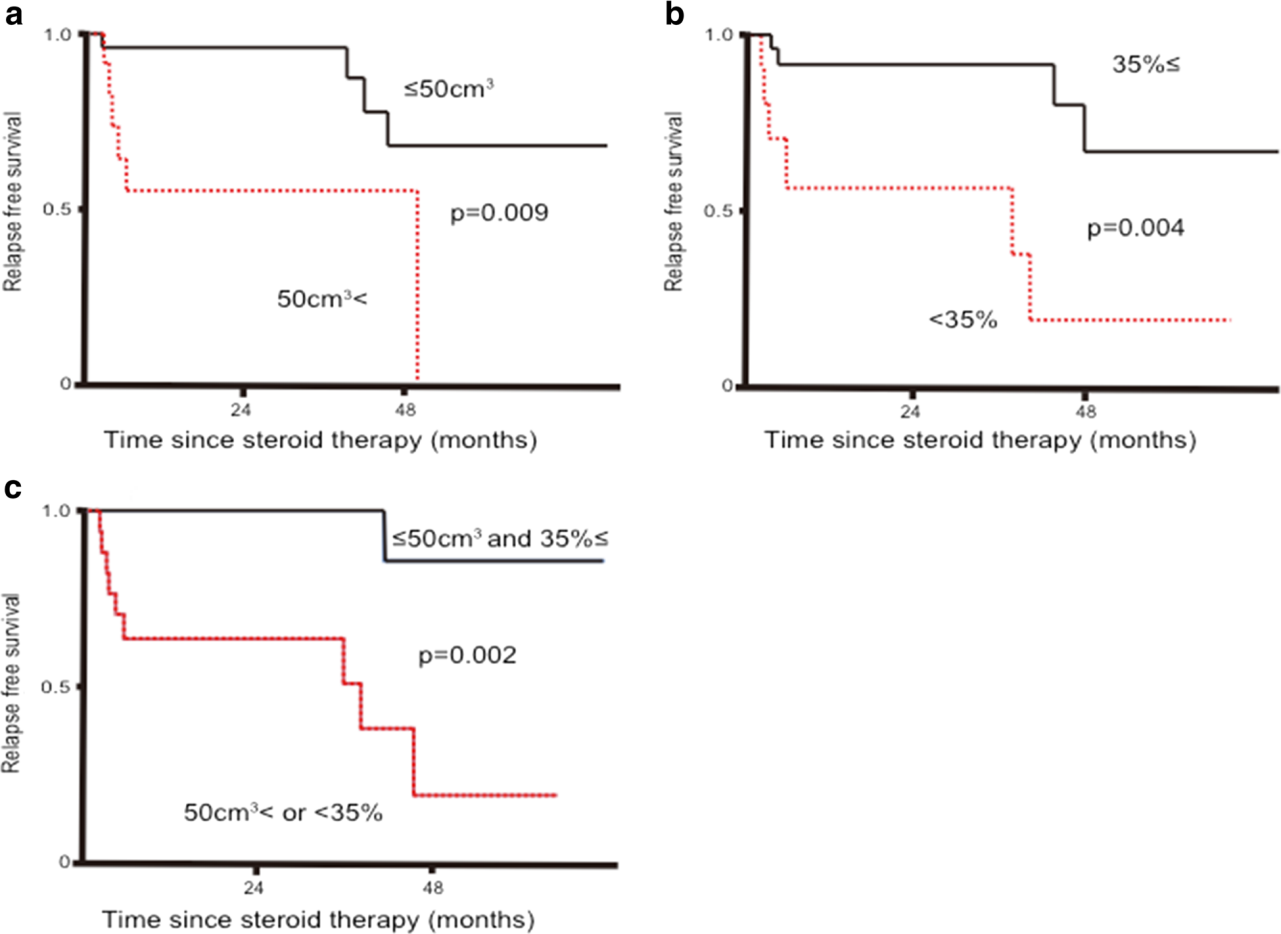
Relapse-free survival rates after steroid therapy. **a** by post-treatment pancreatic volume (cm^3^); **b** by percent reduction in the pancreatic volume (%); **c** by post-treatment pancreatic volume (cm^3^) and percent reduction of pancreatic volume (%). Log-rank test p values are reported

In the ROC curve analysis, the optimal cut-off value of the post-treatment pancreatic volume and the percent reduction in the pancreatic volume for relapse were determined (Fig. 4, Table 4). A post-treatment pancreatic volume 50 cm^3^ < (area under the curve [AUC]: 0.72, sensitivity: 70 %, specificity: 81.8 %, *P* = 0.039) was associated with a significantly high relapse rate in 6 of 10 cases. A percent reduction in the pancreatic volume <35 % (AUC: 0.77, sensitivity: 60 %, specificity: 81.8 %, *P* = 0.009) was also associated with a significantly high relapse rate in 6 of 9 cases. Furthermore, a post-treatment pancreatic volume 50 cm^3^ < or a percent reduction in the pancreatic volume <35 % was associated with a significantly high relapse rate (*P* = 0.002) in 9 of 16 cases. On the other hand, a post-treatment pancreatic volume ≤50 cm^3^ and a percent reduction in the pancreatic volume 35 % ≤ had significantly lower relapse rate, with a relapse in only 1/16 case.

**Table 4:** Relationship between post-treatment pancreatic volume and percent reduction in the pancreatic volume in relapse cases

		Post-treatment pancreatic volume (cm^3^)	
		≤50	50<
Percent reduction in the pancreatic volume (%)	<35	3/6	3/3
	35≤	1/16	3/7

## Discussion

The histologic pattern of type 1 AIP is called lymphoplasmacytic sclerosing pancreatitis, which is characterized by a periductal lymphoplasmacytic infiltrate, storiform fibrosis, and obliterative venulitis [3, 4]. In our study, the pancreatic volume on CT significantly reduced by steroid therapy in all patients. However, when the relapse and non-relapse groups were compared, the change in pancreatic volume after steroid therapy was significantly different. These findings may suggest that the pancreatic volume change after steroid therapy reflects the histological findings in the pancreas. According to the histological findings previously reported, the pancreatic parenchyma was replaced by massive or extensive interlobular fibrosis with lymphoplasmacytic infiltrates to varying degrees in advanced stages of type 1 AIP [14]. However, the pancreatic volumetric blood flow of perfusion CT was attenuated in AIP, which improved after steroid treatment [15]. The pancreatic volumetric blood flow after steroid treatment may reflect the histological disease stage of type 1 AIP. Ko et al. reported that the number of IgG4-positive plasma cells in pancreatic tissue was decreased by steroid treatment, indicating a reduction in inflammation [16]. Nevertheless, changes in the histopathology after steroid treatment for AIP are still unclear. Matsubayashi et al. reported a reduction in the splenic volume by steroid therapy in cases with AIP [17]. In our study, although the volume of the spleen was reduced by steroid therapy, it did not differ between the relapse and non-relapse groups.

In an analysis of 463 patients with AIP among 15 institutes in eight countries, Kamisawa et al. reported that the relapse rate in patients treated with steroid ranged 15–64 % [10]. The relapse rates in Western countries were higher than those in Asian countries (United States, 64 % vs. Japan, 15 %). The difference in the relapse rate was presumably due to the period from administration to cessation of steroid therapy (United States, 3 months vs. Japan, 1–2 years). Indeed, the majority of relapse episodes occurred in steroid-treated subjects following steroid discontinuation compared to those in whom the steroid dose was being tapered or were on steroid maintenance therapy [7, 11]. Continued maintenance treatment with low-dose prednisolone for 6 months to 3 years is also recommended to prevent relapse [7]. Although there is general agreement that long-term steroid therapy is the ideal initial treatment for preventing disease relapse, the incidence of steroid-related side effects is a major concern. Shimizu et al. reported that the cumulative dose of corticosteroids was significantly higher in patients with serious side effects than in those without [18]. In our cases, maintenance steroid therapy was given to all patients, and steroid therapy was discontinued in only 4 patients. Some relapsed patients are treated with an immunomodulator such as azathioprine [9, 11], and these steroid-sparing approaches are attractive for preventing complications from long-term steroid exposure [19–22].

There are some reports regarding the relapse factors in AIP. Diffuse pancreatic swelling was a predictive factor of relapse [11]. The relapse rate of AIP was higher in patients with IgG4-related sclerosing cholangitis (SC) than in those without IgG4-related SC (56.1 % vs. 25.7 %, respectively) [11]. IgG4 seropositivity and jaundice are at a higher risk of relapse, and IgG4 seronegativity have a high likelihood of spontaneous remission [23]. Diffuse pancreatic swelling and proximal biliary involvement are predictive of relapse in type 1 AIP [8], whereas distal biliary involvement was not predictive in cases of type 1 AIP [11]. Additionally, positive staining of the duodenal papilla for IgG4 and a swollen duodenal papilla had a favorable response to steroid therapy [24]. However, factors that may predict relapse have not been well defined, and some are still controversial. Indeed, our study failed to show that the diffuse type IgG4 seropositivity and jaundice were predictive factors of relapse. Moreover, these factors are examined at the time of diagnosis, and they are not intended to reflect the course of treatment.

Over the course of treatment, our findings suggest that early pancreatic volume changes after steroid therapy may be a useful prognostic value, because patients with AIP with a high post-treatment pancreatic volume (50 cm^3^<) or low pancreatic volume reduction (<35 %) showed a significant relapse. Reduction of steroids in these cases must be observed carefully with consideration of immunomodulator use, such as azathioprine.

On the other hand, patients who had a post-treatment pancreatic volume ≤50 cm^3^ and a percent reduction in the pancreatic volume 35 % ≤ had a significantly lower relapse rate. In other words, a low post-treatment pancreatic volume and a high pancreatic volume reduction may predict non-relapse in patients with AIP. Therefore, these measures may be useful for selecting suitable candidates for steroid discontinuation to prevent treatment-related side effects. However, Masuda et al. reported that AIP patients with pancreatic atrophy after steroid therapy have a high incidence of diabetes mellitus [25]. Although our cases did not fit into the definition of pancreatic atrophy proposed by Hirano et al. [26] at the time of CT measurement after steroid therapy, some cases might further reduce their pancreatic volume with a longer period of steroid therapy and develop diabetes. These reports also support the abovementioned idea to discontinue steroid in selected patients.

As is common for studies with retrospective designs, our study has the following limitations: small sample size; not all patients underwent a histological examination; and the steroid treatment regimen, period of steroid therapy, and timing of CT were not uniform. To overcome these limitations, further long-term prospective studies in a larger cohort are needed to examine the relationship between the pancreas volume reduction and relapse in AIP treated with steroids. Nevertheless, we believe that our findings currently present one of the best factors for predicting relapse in patients with AIP.

## Conclusions

Early pancreatic volume reduction on CT after steroid therapy reflects therapeutic effects of steroid and predicts future relapse in patients with type 1 AIP. Reduction of steroids in these cases must be observed carefully with consideration of immunomodulator use.

## Abbreviations

AIP, autoimmune pancreatitis; CT, computed tomography
